# Transfection with thymidine kinase permits bromodeoxyuridine labelling of DNA replication in the human malaria parasite *Plasmodium falciparum*

**DOI:** 10.1186/s12936-015-1014-7

**Published:** 2015-12-02

**Authors:** Catherine J. Merrick

**Affiliations:** Centre for Applied Entomology and Parasitology, Faculty of Natural Sciences, Keele University, Keele, Staffordshire ST55BG UK

**Keywords:** *Plasmodium*, Malaria, BrdU, S-phase, DNA replication, Cell cycle

## Abstract

**Background:**

*Plasmodium falciparum*, the causative agent of severe human malaria, is an early-diverging protozoan whose lifecycle has many unusual features, including its modes of replication. Research on the *Plasmodium* cell cycle, which occurs primarily via schizogony instead of canonical binary fission, has been hampered by a lack of tools and markers that can be transferred from cell cycle studies in model organisms. A common tool used to study DNA replication and the cell cycle in human cells is the labelling of newly-replicated DNA with the modified nucleotide bromodeoxyuridine (BrdU), followed by immunofluorescent detection. *Plasmodium* parasites, however, do not incorporate BrdU because they rely only on *de novo* synthesis of pyrimidines and do not salvage thymidine analogues like BrdU for conversion into nucleotides.

**Methods:**

Analysis of biochemical pathways in *Plasmodium* indicated that the absence of the enzyme thymidine kinase (TK) may be the only impediment to BrdU incorporation in this organism. A TK gene from *Herpes simplex* was, therefore, introduced into the *Plasmodium falciparum* 3D7 strain and the effect on BrdU labelling was assessed by enzyme-linked immunosorbent assay and immunofluorescence microscopy.

**Results:**

Introduction of a TK gene produces parasites that can indeed incorporate BrdU. This forms a sensitive indicator of DNA replication, which can be detected by both quantitative and qualitative assays on either a population level or a single-cell level. *Plasmodium falciparum*, when expressing TK, becomes unusually sensitive to BrdU toxicity.

**Conclusions:**

BrdU labelling represents a significant new tool for investigating DNA replication and the cell cycle in *Plasmodium*.

**Electronic supplementary material:**

The online version of this article (doi:10.1186/s12936-015-1014-7) contains supplementary material, which is available to authorized users.

## Background

Human malaria caused by *Plasmodium* parasites gives rise to widespread morbidity and more than half a million deaths each year [1]. New methods of malaria control, including novel anti-malarial drugs, are urgently needed and their development could be informed by a better understanding of the basic biology of the causative parasite: an unusual protozoan with a complex lifecycle. *Plasmodium* lives primarily intracellularly in its two hosts, the human (or other vertebrate host) and the mosquito, where it undergoes distinct modes of both sexual and asexual replication. Modes of cell division differ at the different lifecycle stages, but *Plasmodium* does not divide by binary fission, in fundamental contrast to the normal cell cycle of both its hosts. Instead, it divides primarily by schizogony: this is the division mode for all the lifecycle phases that occur in the human host, both inside hepatocytes and inside erythrocytes. In schizogony, multiple rounds of DNA replication occur inside a single cell prior to cytokinesis. This complicates the interpretation of the *Plasmodium* cell cycle in terms of canonical phases: gap 1 (G1), DNA synthesis (S), gap 2 (G2) and mitosis (M) [2–4]. Like any difference between the basic biology of host and parasite, schizogony presents a possible drug target. However, many aspects of the *Plasmodium* cell cycle are poorly understood, in stark contrast with the extensively studied conventional eukaryotic cell cycle.

Research on this very basic aspect of *Plasmodium* biology has been hampered by a lack of tools and markers that can be transferred directly from cell cycle studies in model organisms. For example, the cyclins and cyclin dependent kinases (CDKs) that are central regulators of cell cycle phases in all eukaryotes from yeast to human remain relatively poorly characterized in *Plasmodium* [5, 6]. Many chemical synchronizing agents do not work well on blood-stage parasites [7, 8], and flow cytometric monitoring of S-phase via cellular DNA content is complicated by multiple asynchronous rounds of replication within each schizont. As a result, determining exactly what phase of the cell cycle a parasite is in, or when it starts and finishes S-phase, is largely limited to assessing the morphology of the parasite by microscopy, as it develops from a pre-replicative ring stage into a replicative trophozoite stage and then into a schizont stage, in which individual nuclei become visible inside the parent cell.

The incorporation of BrdU into actively-replicating DNA, followed by immunofluorescent detection with anti-BrdU antibodies, has long been a workhorse assay in mammalian cells, detecting cells in S-phase swiftly and sensitively. In fact, in the large nuclei of mammalian cells, distinct patterns of replication foci can be labelled at different stages of S-phase, allow the finer distinction of cells that are in early, mid or late S-phase [9].

Attempts were made more than two decades ago to adapt the BrdU labelling technique for *Plasmodium* (particularly because at this time, quantitative monitoring of parasite replication otherwise required the laborious incorporation of tritiated hypoxanthine, followed by scintillation counting). In 1988, an initial report was published on BrdU incorporation into *Plasmodium*, showing positive results with an enzyme-linked immunosorbent assay (ELISA) [10]. Two further reports on this have also appeared in later years [11, 12]. By contrast, it has also been reported that *Plasmodium* parasites do not incorporate BrdU and that the absorbance of light by haemozoin, which accumulates inside schizonts, can be taken for *bona fide* labelling when measured only by ELISA [13]. *Plasmodium* is well known to rely on *de novo* synthesis of pyrimidines and does not therefore salvage thymidine analogues like BrdU for conversion into nucleotides [14–18].

The intra-erythrocytic parasite expresses a combination of membrane channels that should allow BrdU to cross all three membranes enclosing the parasite: the erythrocyte membrane, the parasitophorous vacuole membrane and the parasite plasma membrane [19–23]. There is also a tubulovesicular network that can bring small metabolites into close proximity with the parasite plasma membrane [24, 25]. Therefore, the only impediment to BrdU incorporation in *Plasmodium* species may be biochemical: the absence of the enzyme thymidine kinase (TK), which converts thymidine, and also its analogs such as BrdU, from a deoxynucleoside into a deoxynucleotide which can subsequently be incorporated into DNA. This same problem has been overcome in *Saccharomyces cerevisiae* by expressing a viral TK transgene [26]. With the advent of molecular genetics in malaria parasites, this is now similarly possible in *P. falciparum*, in which TK from *Herpes simplex* virus has been used as a negative selectable marker, making the parasites sensitive to pro-drug nucleoside analogues such as ganciclovir [27].

This report shows that TK-expressing parasites can indeed be labelled with BrdU. Labelling can be detected by ELISA and also by immunofluorescence microscopy in single cells: evidence that was never shown in prior reports of BrdU labelling in wildtype parasites. In parallel assays, wildtype parasites remain unlabelled, just as reported in 1991 by Janse et al. [13]. At a population level, BrdU labelling measured by ELISA is a much more sensitive indicator of DNA replication than the standard malaria SYBR Green I fluorescence (MSF) assay [28]. Interestingly, TK-expressing parasites are much more sensitive to BrdU toxicity than mammalian cells. Nevertheless, BrdU labelling, when used in short-term endpoint assays, will represent a significant new tool for investigating DNA replication and the cell cycle in this unusual and medically-important parasite.

## Methods

### Parasites and parasite culture

*Plasmodium falciparum* 3D7 parasites were obtained from MR4 (mr4.org). Parasites were cultured in human O^+^ erythrocytes at 4 % haematocrit in RPMI 1640 supplemented with 0.25 % albumax (Invitrogen), 5 % heat-inactivated human serum and 0.25 % sodium bicarbonate, using standard procedures [29]. Synchronized ring-stage parasites were produced by treatment with 5 % sorbitol [30], or by Percoll separation of schizont stages, followed by re-invasion into fresh erythrocytes [31].

### Generation and characterization of TK-expressing parasites

All transgenic parasites carried versions of the pHTK double-selectable gene-targeting plasmid ([27], available from MR4), containing various different gene-targeting sequences cloned from the 3D7 genome, then transfected into 3D7 parasites using standard procedures [32]. Southern blotting using the AlkPhos Direct Labelling and Detection system (GE Healthcare) was used to confirm that the parasite lines ‘+TK’ and ‘+TK(B)’ were carrying the pHTK plasmid episomally, and that ‘+TK(int)’ was carrying a genome-integrated copy. Plasmid copy number in each +TK parasite line was determined by quantitative PCR using a StepOne Plus machine (Applied Biosystems) and the SensiFAST SYBR Hi-ROX kit (Bioline), on genomic DNA extracted from each parasite line via the QIAamp DNA blood mini kit (Qiagen). Primers for the TK gene, used at a final concentration of 125 nM, were: AGA AAA TGC CCA CGC TAC TG (forward) and CTC GAC CAG GGT GAG ATA TC (reverse). Primers for two single-copy housekeeping genes used as controls, PF3D7_0717700 (seryl-tRNA synthetase) and PF3D7_1246200 (actin), are previously described [33]. Cycling conditions were 50 °C 2 min, 95 °C 3 min, 40cycles of 95 °C 15 s, 54 °C 40 s, 60 °C 1 min. The Relative Copy Number (RCN) of the TK gene was calculated as 2^ΔCt^, where ΔCt was relative to the average Ct of the two control genes. All PCR reactions were carried out in triplicate.

### Malaria SYBR Green I fluorescence (MSF) assay

The BrdU (Sigma) used in all assays was dissolved in water as a 50 mM stock, aliquoted, frozen at −20 °C and thawed as required for dilution into culture medium. MSF assays for parasite growth were carried out essentially as previously described [28], plating synchronized trophozoite-stage parasites at 0.5 % parasitaemia and 2 % haematocrit in a 96-well format containing threefold serial dilutions of BrdU and then incubating for 48 h. In BrdU pulse assays, aliquots of parasite culture were incubated in BrdU for the required period, then washed twice in fresh culture medium before plating. When ring-stage parasites were used in pulse assays, a sorbitol treatment (as for synchronization) was also used prior to the two washes, to eliminate any parasites that had progressed to trophozoite stage during the pulse-label: this ensured that only parasites exposed as rings were subsequently plated.

To read these assays, the resultant cultures were mixed with MSF lysis buffer and SYBR Green I fluorescence was measured using the GloMax multidetection system (Promega). Percentage parasite growth was calculated relative to the fluorescence readouts from the control culture, incubated without BrdU (100 % growth), and the culture incubated with the maximum concentration of BrdU (0 % growth). All assays were carried out in technical triplicate and representative datasets are presented from at least 2 biological replicates. GraphPad Prism v.6.0 (GraphPad software Inc.) was used to plot growth inhibition curves via non-linear regression fitting, and thus to calculate 50 % inhibitory concentration (IC50) values.

### 3-(4,5-dimethylthiazol-2-yl)-5-(3-carboxymethoxyphenyl)-2-(4-sulfophenyl)-2H-tetrazolium (MTS) assay

The MTS assay for viability of human cells was carried out using the MCF-7 breast cancer cell line, cultured in Eagle’s MEM. Trypsinized cells were plated in 96-well format at 2 × 10^4^ cells/well, allowed to attach for 3 h, then exposed to fivefold serial dilutions of BrdU for approximately 1 cell cycle (40 h), as per the MSF assay on parasites. The assay was read using the CellTiter 96 Aqueous One Solution Cell Proliferation kit (Promega) in the GloMax multidetection system (Promega).

### Parasite life-cycle assay

Early ring-stage parasites, synchronized using Percoll, were exposed to 1 mM, 100 nM or 0 BrdU and their development was followed by blood smears, stained using the Hemacolor staining kit (Merck Millipore), at 8 h intervals over the subsequent cell cycle. 100 parasites were classified for their developmental stage at each timepoint.

### ELISA

Parasites were prepared for ELISA by releasing from erythrocytes using 0.15 % saponin/PBS, washing and re-suspending in PBS, then plating in triplicate in 96-well format at either 2 × 10^5^ or 1 × 10^6^ parasites/well. Plates were allowed to air-dry, fixed for 5 min in 4 % formaldehyde/PBS and for 2 min in 50/50 methanol/acetone, then washed 3 times in PBS and blocked for 1 h in 1 % BSA/PBS. All wells were treated for 3 h at 37 °C with mouse monoclonal anti-BrdU antibody BU-1 plus nuclease (Amersham, kit RPN202), washed three times in PBS, then treated for 1 h with anti-mouse HRP-conjugated secondary antibody (Dako) diluted 1:5000 in BSA/PBS. After three further washes in PBS, assays were developed using the Single-Component TMB Peroxidase EIA Substrate Kit (BioRad), stopped with 0.6 N sulphuric acid and then read using the GloMax multidetection system (Promega). In parallel with the plating of parasites for ELISA, aliquots of the same suspension of parasites were mixed with MSF lysis buffer for an MSF-type assay, to confirm equal loading of parasite genomes across ELISA plates. All assays were carried out in triplicate.

### Immunofluorescence

Parasites were prepared for immunofluorescence as air-dried blood smears, fixed for 5 min in 4 % formaldehyde/PBS, then for 2 min in 50/50 methanol/acetone, then washed 3 times in PBS and blocked for 30 min in 1 % BSA/PBS. Slides were treated for 1 h with mouse monoclonal anti-BrdU antibody BU-1 plus nuclease (Amersham, kit RPN202), washed three times in 1 % BSA/PBS, treated for 1 h with anti-mouse Cy3-conjugated secondary antibody (Stratech Scientific) diluted 1:5000 in BSA/PBS, then washed again three times with BSA/PBS. 2 mg ml^−1^ 4′,6-diamidino-2-phenylindole (DAPI) was included in the second wash. Slides were mounted in 50/50 PBS/glycerol and examined using a Zeiss Axio Scope A1.

## Results

### Expression of thymidine kinase allows *P. falciparum* parasites to incorporate BrdU into genomic DNA

**Fig. 1:**
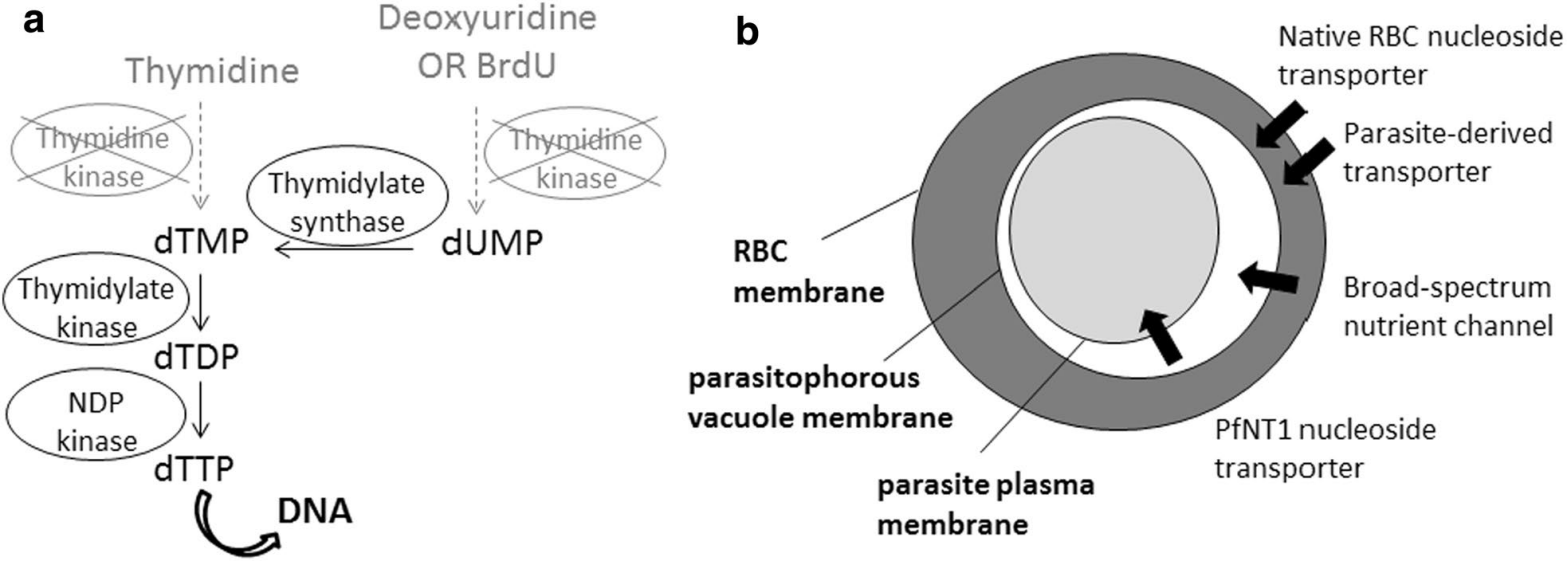
Schematic of nucleotide metabolism and transport pathways in *P. falciparum*. **a** Enzymes required for the metabolism of pyrimidine nucleosides to nucleotides for incorporation into DNA. *Plasmodium* species lack thymidine kinase but contain all the other enzymes shown downstream. **b** Schematic showing the route by which modified nucleosides like BrdU could enter intra-erythrocytic parasites. *RBC* red blood cell, *PfNT1* *P. falciparum* nucleoside transporter 1

Biochemical pathways in *P. falciparum* have been extensively characterized by homology searches within the sequenced genome and/or experimentally [34]. Analysis of these pathways shows that the genome does not encode a TK enzyme, but does encode all the required enzymes for downstream conversion of nucleosides into nucleotides (Fig. 1a). Therefore, the only impediment to BrdU incorporation at the biochemical level should be the lack of a TK enzyme, since intra-erythrocytic parasites express a combination of channels that should allow nucleoside analogs like BrdU to enter the parasite (Fig. 1b).

**Fig. 2:**
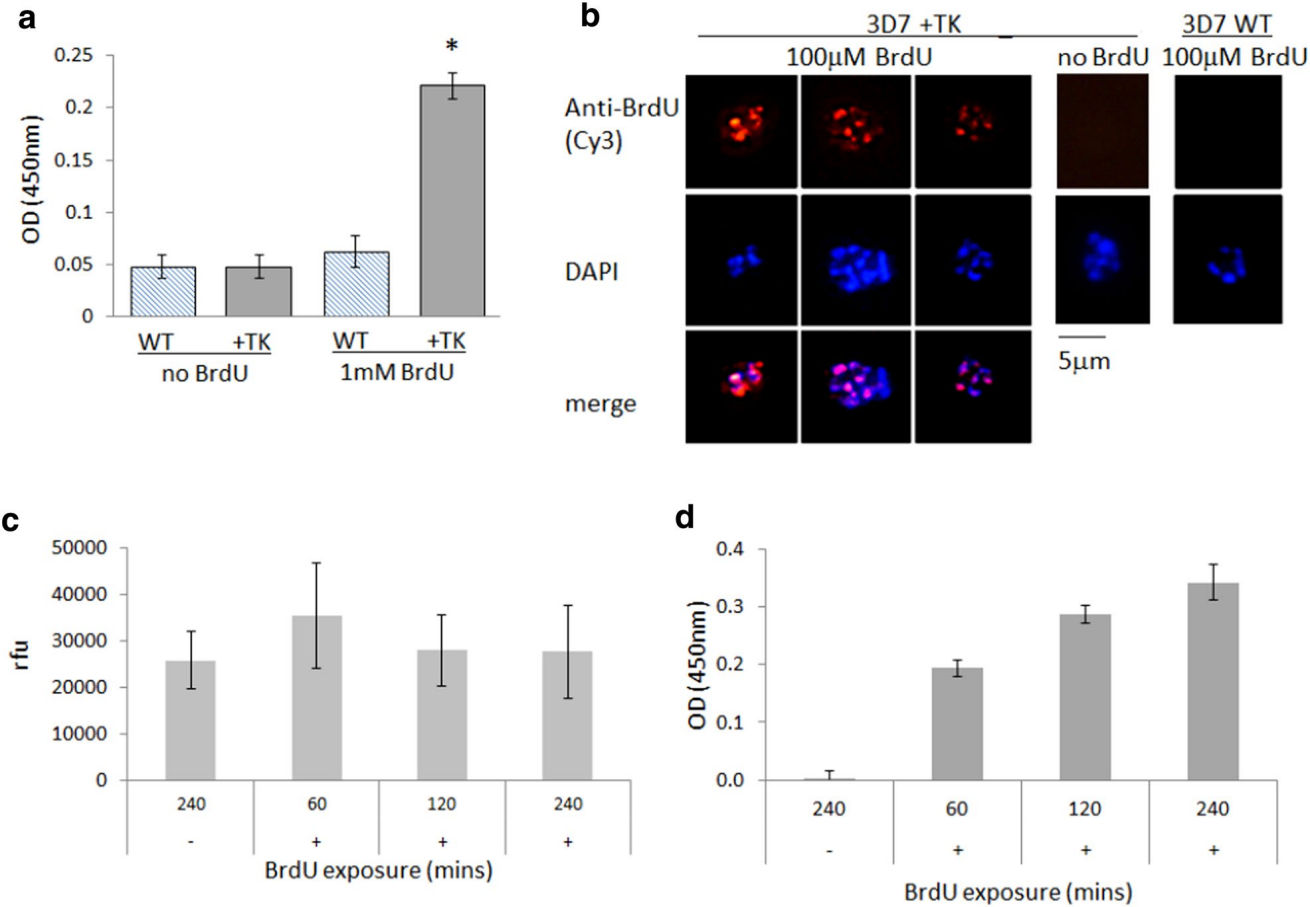
TK-expressing *P. falciparum* can incorporate BrdU, which is a sensitive indicator of DNA replication. **a** ELISA on wild-type (WT) and +TK parasites (2 × 10^5^ parasites/well) after exposure to 1 mM BrdU for 4 h at the trophozoite stage. *Error bars* show standard deviation of triplicate readings; star indicates the only significantly differing dataset (One-way ANOVA, p < 0.0001). **b** Immunofluorescence assay on (WT) and +TK parasites exposed to 100 mM BrdU for 12 h at trophozoite stage. **c** SYBR Green I staining of +TK parasites after exposure to no BrdU (−) or to 100 mM BrdU (+) for 1, 2 or 4 h. Parasites were released from erythrocytes and suspended in PBS [as for the ELISA assay shown in (**a**)], then plated with MSF lysis buffer at 1 × 10^6^ parasites/well. The increase in DNA content over 3 h is not detectable by this MSF-type assay. *Error bars* show standard deviation of triplicate readings. rfu, relative fluorescence units. **d** ELISA on the same +TK parasites as in (**c**), plated at 1x10^6^ parasites/well after exposure to no BrdU (−) or to 100 mM BrdU (+) for 1, 2 or 4 h. Background signal, obtained from the same parasites incubated without BrdU, has been subtracted from all readings, and *error bars* show standard deviation of triplicate readings. Figure **c** confirms that approximately the same numbers of parasites were plated in all cases

A TK enzyme was, therefore, expressed in *P. falciparum* parasites by transfecting the standard laboratory strain, 3D7, with the pHTK plasmid [27]. This plasmid carries the human dihydrofolate reductase (h*DHFR*) gene as a positive selectable marker and a *Herpes simplex* TK gene as a negative selectable marker to facilitate the generation of genetic knockouts by double homologous recombination. A parasite line carrying this plasmid as an episome (‘+TK’) was able to incorporate BrdU into actively replicating trophozoite parasites, as demonstrated using both immunofluorescence and ELISA (Fig. 2a, b). Wildtype 3D7 parasites exposed to BrdU in parallel did not show detectable levels of labelling, even at very high exposures of up to 1 mM for several hours (Fig. 2a, b), thus showing that the TK transgene was absolutely required for BrdU to be converted into a nucleotide and incorporated into genomic DNA.

### BrdU is a more sensitive indicator of DNA replication than SYBR Green I DNA dye

BrdU labelling allows newly-replicated DNA to be distinguished from existing DNA, which is not possible with standard DNA-intercalating dyes such as 4′,6-diamidino-2-phenylindole (DAPI) or SYBR Green I. In addition, BrdU labelling offers increased sensitivity compared to SYBR Green I, which is the DNA dye used in the standard MSF assay for parasite growth [28]. The MSF assay measures the increase in DNA content as a population of parasites progresses through at least one replicative cycle, generating an average of 16–32 new genomes per parasite per cycle. The MSF assay gives a broadly quantitative measure of DNA content but it has a relatively low dynamic range and high signal-to-noise ratio [35]. To compare this with the sensitivity of BrdU labelling, +TK parasites at mid-trophozoite stage (~30–36 h post invasion) were exposed to BrdU for 1–4 h. Four hours should cover approximately one round of genome replication if a parasite produces 16–32 daughter parasites via 4–5 successive genome replications during its 16–20 h trophozoite stage [3]. DNA replication during a 4-h period was not reliably detectable by SYBR Green I in an MSF assay, but was clearly detectable by anti-BrdU ELISA (Fig. 2c, d).

### Parasites expressing thymidine kinase become sensitive to BrdU toxicity

The BrdU labelling technique is primarily used in short-term endpoint assays, but in mammalian cells it can also be used over several cell cycles, and even in vivo, with BrdU being added to the drinking water of mice to monitor the turnover of cellular compartments such as haematopoietic and intestinal cells over weeks or months [36]. BrdU is thus considered to be a relatively non-toxic DNA label in mammalian cells, despite the fact that it was originally developed as an anti-cancer agent to disrupt DNA synthesis, and has been tested—largely without success—in clinical trials as a radio-sensitizing agent. In a single-celled parasite like *Plasmodium*, this sort of long-term assay is unlikely to be required, but the usefulness of BrdU labelling might nevertheless be limited if acutely toxic levels were required to label parasites. Therefore, the toxicity of BrdU on *P. falciparum* was tested in a standard MSF assay.

**Fig. 3:**
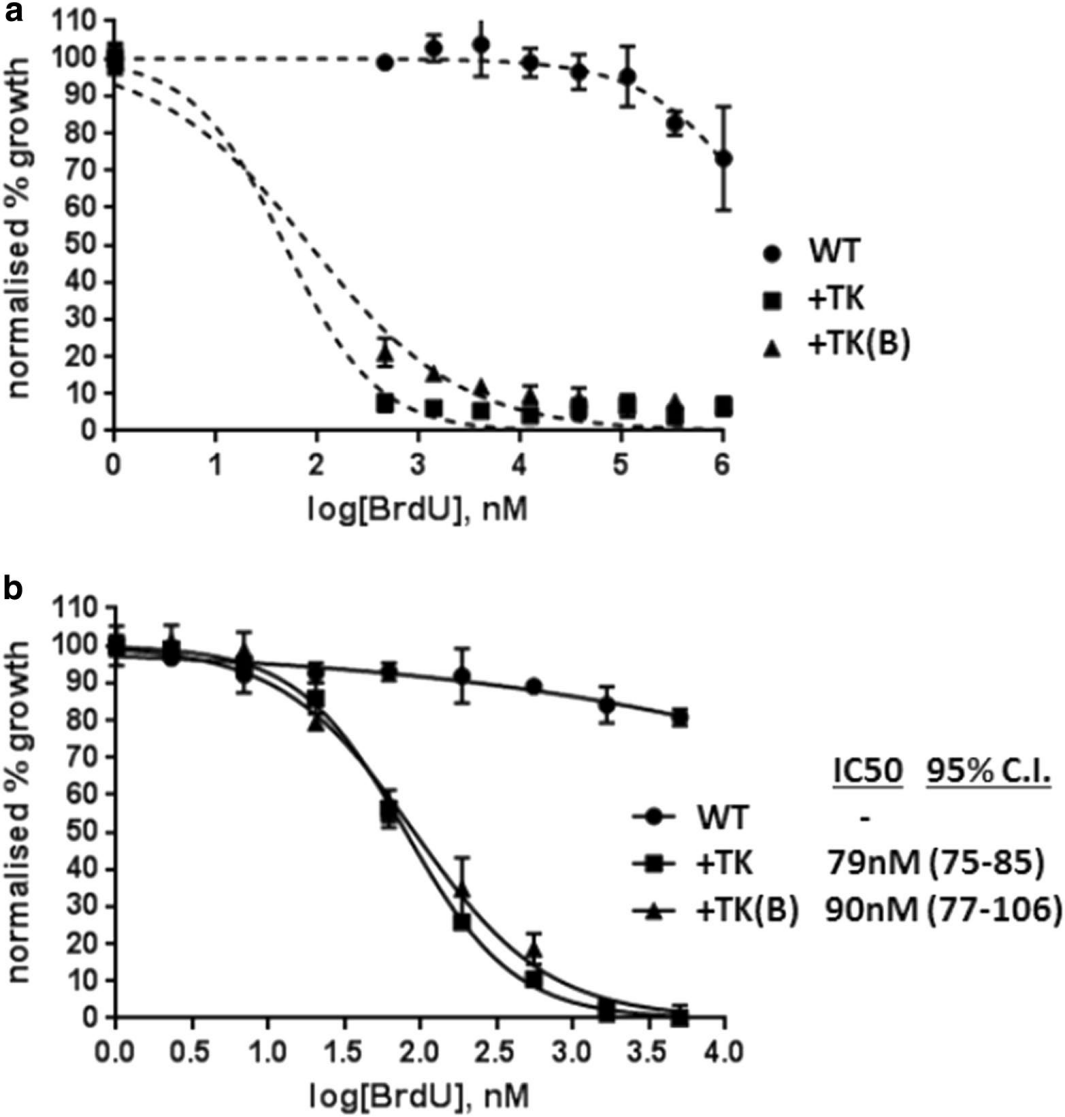
TK-expressing parasites become sensitive to BrdU toxicity. **a** MSF assay on WT and two different TK-expressing parasite lines, +TK and +TK(B), over a BrdU range of 1–0.46 mM. *Error bars* show standard deviation of triplicate readings. **b** MSF assay on the same parasites as in (**a**), over a range of 5–2.3 nM BrdU. IC50 values were calculated with GraphPad Prism software, 95 % confidence intervals are given in *parentheses*

Wildtype 3D7 parasites were essentially insensitive to BrdU, even at very high exposures of up to 1 mM over a 48-h growth cycle (Fig. 3a). By contrast, +TK parasites were surprisingly sensitive, with a 50 % growth-inhibitory concentration (IC50) below 100 nM (Fig. 3b). Two independent parasite lines, carrying different versions of the TK plasmid at different average copy-numbers (Additional file 1A), were tested to ensure that the presence of the TK gene was consistently responsible for this effect. IC50 values of 79 and 90 nM were measured in the two independent lines, confirming that +TK parasites behave consistently and also that the TK gene copy number has no apparent effect on BrdU sensitivity.

Several precautions were taken to ensure that the toxicity of BrdU in +TK *P. falciparum* lines was not artefactual. Firstly, an analogous assay for cell viability was carried out on a human cell line, MCF-7, using the same BrdU stock, to ensure that it was not toxic to human cells. These cells, as expected, showed no acute death when exposed to up to 1 mM BrdU over one cell cycle (Additional file 1B). All assays, on both human and *Plasmodium* cells, were incubated in the dark at all times to reduce the risk of DNA damage caused by photo-activation of BrdU in DNA, because this compound is known to be UV-sensitizing as well as radio-sensitizing [37]. Finally, assays were carried out in both the presence and absence of the positive-selection agent for the pHTK plasmid, WR99210. This drug inhibits the dihydrofolate reductase activity of the *P. falciparum* bifunctional dihydrofolate reductase-thymidylate synthase enzyme (*Pf*DHFR-TS), but does not affect the human DHFR enzyme [32]. Therefore, WR99210 selects for the maintenance of plasmids carrying the h*DHFR* gene, because maintaining dihydrofolate reductase activity is essential for parasite growth. Human DHFR is well-established to be a fully effective substitute for the *P. falciparum* enzyme [32] and parasite lines carrying such plasmids grow stably, without impairment, on WR99210 selection; nevertheless, it remained possible that interfering with this pathway in the presence of a non-natural brominated base might somehow affect the toxicity of BrdU. Therefore, assays were carried out using a parasite line that carried the pTK plasmid stably integrated into the genome via a single homologous recombination event, to avoid rapid episome loss in the absence of drug selection. WR99210 was removed from the parasites in the growth cycle preceding the MSF assay, and no difference in the IC50 of BrdU was seen in the presence or absence of WR99210 (Additional file 1C).

### BrdU causes schizont-stage arrest in TK-expressing parasites

**Fig. 4:**
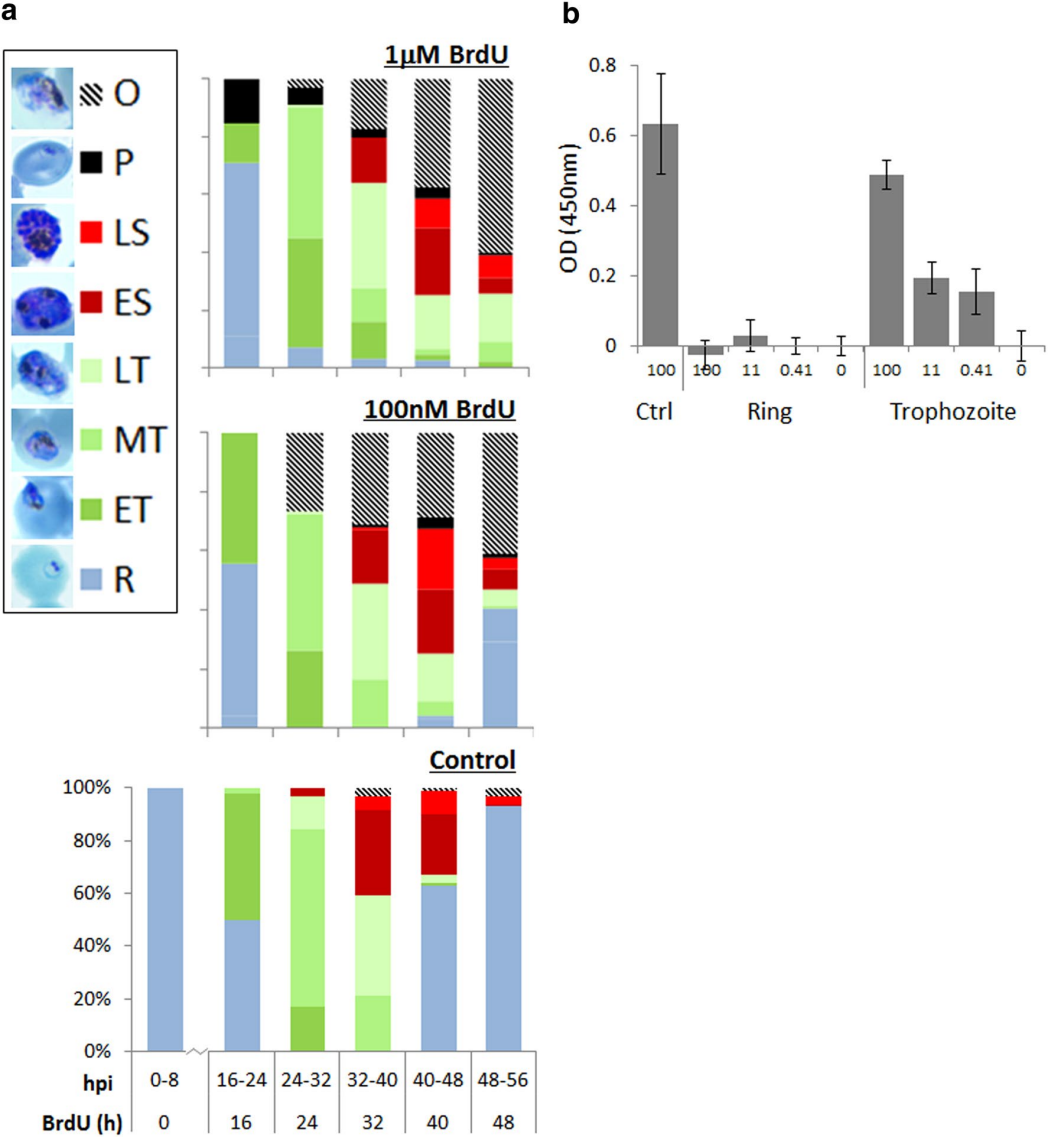
BrdU causes schizont-stage arrest in TK-expressing parasites. **a** Developmental stages of +TK parasites assessed at 8 h intervals over a single growth cycle in the presence of 0, 100 nM or 1 mM BrdU. 100 parasites were counted at each timepoint. *Photographs* show representative parasite morphology at each stage: *R* ring, *ET* early trophozoite (parasite less than half the width of host cell), *MT* middle trophozoite (parasite more than half the width of, but not entirely filling, host cell), *LT* late trophozoite (parasite filling all or nearly all of host cell), *ES* early schizont (nuclear masses visible within parasite), *LS* late schizont (defined merozoites visible), *P* pyknotic (parasite stained as a dense shrunken intracellular dot without clear morphological features), *O* parasite outside erythrocyte, *hpi* hours post invasion. **b** ELISA on +TK parasites, plated at 1 × 10^6^ parasites/well, after ring-stage exposure to 0, 0.41, 11 or 100 mM BrdU for 4 h. Parasites were either harvested immediately after washing out the BrdU, as rings, or cultured until the late-trophozoite stage and then harvested for ELISA. Control (ctrl) parasites were cultured to the trophozoite stage before exposing to 100 mM BrdU for 4 h at this stage and then harvesting immediately. The background signal, obtained from parallel samples incubated without BrdU, has been subtracted from each set of readings. *Error bars* show standard deviation of triplicate readings

The standard MSF assay used in Fig. 3 simply measures overall growth inhibition over a 48-h growth cycle from trophozoites to daughter trophozoites. To investigate the exact stage of the *Plasmodium* cell cycle at which BrdU toxicity becomes apparent, highly synchronized ring-stage +TK parasites (Fig. 4a, 0–8 h) were treated with either 0, 100 nM (~IC50) or 1 mM (~IC95) BrdU and morphology was assessed at regular intervals over the subsequent growth cycle (Fig. 4a). Parasites appeared to develop normally through the pre-replicative ring stage and most of the trophozoite stage, although some trophozoites exposed to BrdU did begin to appear outside erythrocytes (where they presumably become inviable). The majority of parasites, however, progressed to the late trophozoite or early schizont stage. BrdU-exposed parasites then generally failed to release merozoites and reinvade: in 1 mM BrdU, there was a complete lack of reinvasion and only arrested, burst or exoerythrocytic parasites remained by the time the control population had produced the next generation of rings. At 100 nM BrdU, reinvasion did occur, but only at 49 % of the control rate (consistent with this representing an approximately IC50 exposure). This pattern closely resembles the parasite killing effect of WR99210, which prevents the completion of DNA synthesis by disrupting the supply of nucleotide precursors. WR99210 similarly causes late-stage parasites to swell, burst and fail to reinvade.

**Fig. 5:**
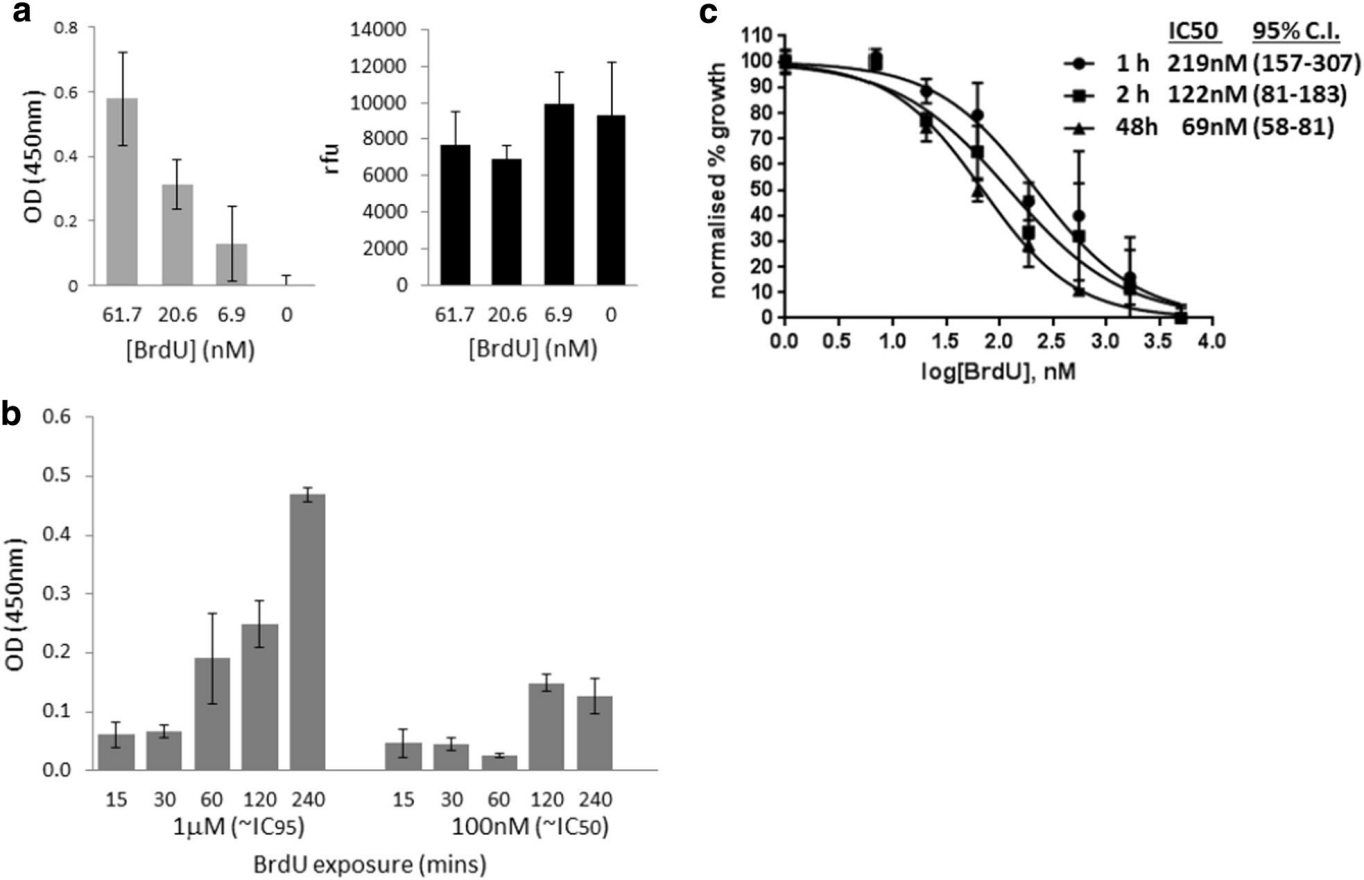
Toxicity does not preclude the use of BrdU as a short-term labelling agent in *P. falciparum.* **a** ELISA on +TK parasites, plated at 1 × 10^6^ parasites/well, after 48 h exposure to the three lowest concentrations of BrdU used in the MSF assay shown in Fig. 3b. Background signal, obtained from the sample incubated without BrdU, has been subtracted from all readings. *Error bars* show standard deviation of triplicate readings. A parallel MSF-type assay on the same parasites shows that DNA content is similar in all samples, confirming that within 48 h, severe growth inhibition has not occurred at these sub-IC50 exposures. **b** ELISA on +TK parasites (1 × 10^6^ parasites/well), after pulses of BrdU from 15 min to 4 h, applied at the trophozoite stage. The background signal, obtained from parallel samples incubated without BrdU, has been subtracted from each set of readings. *Error bars* show standard deviation of triplicate readings. **c** MSF assay on +TK parasites after 1 or 2 h pulses of BrdU (5 mM to 2.3 nM) at trophozoite stage, followed by washing and growth in fresh medium for 48 h. Parasites were also kept in BrdU for the full 48 h, as in Fig. 3b, for comparison. *Error bars* show standard deviation of triplicate readings. IC50 values were calculated with GraphPad Prism software, 95 % confidence intervals are given in *parentheses*

These observations are consistent with a specific toxic effect occurring in schizonts after BrdU has been incorporated into genomic DNA. If the ring stage, prior to the onset of DNA replication, is genuinely insensitive, then parasites exposed to a pulse of BrdU as rings might be expected to develop normally through the subsequent replicative stages. However, a 4-h pulse of BrdU applied in the ring stage still appeared to be toxic if parasites were then allowed to progress through the subsequent replicative stage and attempt reinvasion (data not shown). This raised the question: does the presence of brominated nucleosides or nucleotides have another (morphologically undetectable) effect on the ring stage itself? Or does ring-stage exposure simply load BrdU into the nucleotide pools later used for DNA replication?

Parasites were exposed to 4-h pulses of BrdU as rings, then washed and either harvested immediately or as late trophozoites in the replicative stage following exposure. No BrdU was detectable by ELISA in the ring-stage parasites, but after progression through the trophozoite stage, the parasites did become labelled with BrdU almost as strongly as when exposed during the trophozoite stage itself (Fig. 4b). This does not exclude any specific effect of BrdU on the ring stage, but it does suggest that ring-stage exposure to BrdU can indeed load the nucleotide pool that is later used by the trophozoite to replicate the genome during S-phase.

### The unusual sensitivity of *Plasmodium falciparum* to BrdU does not preclude the use of BrdU as a short-term labelling agent

For most assays exploring S-phase dynamics, BrdU labelling for a full 48-h growth cycle is not necessarily required, and Figs. 2b and 4a both show that parasites develop quite normally through to the latest stages of S-phase even when exposed to BrdU at levels above the 48-h IC50. Nevertheless, if long labelling periods *are* required, BrdU can clearly be detected by ELISA in a parasite population exposed for 48 h to BrdU levels well below the IC50 (Fig. 5a). Labelling can also be detected after much shorter pulse-labels of trophozoite parasites (Fig. 5b): certainly as little as 1 h at 1 mM, or 2 h at 100 nM. Short pulse labels are somewhat less toxic than continuous exposure for a whole 48 h: for example, the 48-h IC50 following a 1-h pulse of BrdU was measured at 219 nM (Fig. 5c): three times higher than the IC50 for continuous exposure.

In conclusion, the BrdU labelling technique could prove useful for many experiments on DNA replication in *P. falciparum,* despite the unusually toxic effect of BrdU in this organism.

## Discussion

The technique reported here will allow BrdU labelling of replicating DNA to be used in any *Plasmodium* parasite that is transfected with a TK gene. The technique is fundamentally different from the use of standard DNA dyes to detect the genomic contents of parasites, because it can distinguish new DNA synthesized during any defined labelling period from pre-existing DNA.

While developing this technique, it became apparent that *P. falciparum* parasites expressing a TK transgene are unusually sensitive to BrdU toxicity. The reason for this is unclear, but certainly has to do with the non-natural ability to metabolize BrdU into a nucleotide—and probably to incorporate it into genomic DNA during S-phase—because parasites lacking the TK gene are entirely insensitive. Notably, the toxic effect of high-level BrdU means that it could be as effective as ganciclovir in negative selection of TK-carrying genetic recombinants. This may be particularly advantageous if it reduces the ‘bystander effect’ seen with ganciclovir, in which neighbouring cells are killed via diffusion of toxic metabolites [27].

BrdU is not universally toxic in organisms that do not naturally possess TK activity: *S. cerevisiae* also lacks TK, but overexpression of a TK transgene allows this yeast to incorporate BrdU without overt toxicity [26]. Several unusual features of *P. falciparum* biology may account for its inability to survive well with BrdU-substituted DNA. This parasite has one of the most AT-rich genomes ever sequenced at 80.6 % [38], so the potential density of BrdU substitutions for thymidine bases is unusually high. However, similarly biased genomes do exist, notably in *Dictyostelium discoideum* (77.6 % A/T [39]), and although BrdU may inhibit the developmental cycle of this organism [40, 41], vegetative amoebal growth does not appear to be highly sensitive to BrdU. Indeed, an IC50 of 75 mg ml^−1^ (0.24 mM) appears in one report [42], which is more than a thousand-fold higher than the IC50 for TK-expressing *P. falciparum,* and BrdU labelling experiments at very high exposures have been used in *D. discoideum* without reported toxicity [43].

A second possibility relates to the near-absence of CpG methylation in the *P. falciparum* genome (this modification was long thought to be entirely absent [44]; it has recently been detected, but at unusually low levels [45]). It may be that the parasite has a diminished ability to tolerate, replicate through or transcribe through modified pyrimidine bases, and therefore experiences replicative or transcriptional problems upon encountering brominated bases.

Thirdly, the unusual nature of schizogony may present a particularly severe problem for BrdU-substituted DNA, as suggested by the specific cell cycle arrest observed at the schizont stage. During schizogony, the parasite has the topologically challenging task of separating dozens of genomes into individual merozoites, without apparent chromosome condensation, and BrdU-substituted genomes may disturb this process, particularly if they cause, or are recognized as, DNA damage. In human cells, BrdU does cause DNA damage and the activation of cell cycle checkpoints, albeit only at much higher levels. For example, a week-long exposure of one lung cancer cell line to 10 mM BrdU was reported to elicit features of a DNA damage response, cell cycle arrest, and eventually a senescence-like phenotype without cell death [46]. Little is known about DNA damage checkpoints in *Plasmodium*; in fact this is one very attractive area to which to apply this new technique.

The BrdU labelling technique will also allow the *Plasmodium* cell cycle to be studied at unprecedented temporal resolution. This is of considerable interest for characterizing the dynamics of intra-erythrocytic schizogony, and arguably even more so for examining the very rapid process of male gametogenesis [47] (objectives that we are now actively pursuing). In particular, the dynamics of successive replicative cycles during schizogony remain unexplored. This report has clearly showed that parasites can pre-load their nucleotide pools with BrdU during the ring stage and that this cannot be ‘washed out’, presumably because the cell, although permeable to nucleosides, is not equally permeable to phosphorylated, and therefore charged, nucleotides. Thus, significant levels of BrdU labelling (and associated toxicity) are carried over into the replicative trophozoite stage. In human cells, nucleotide pools during S-phase can be rate-limiting for DNA replication: the pool increases sharply at S-phase entry and then throughout S-phase, resulting in replication rates increasing 2 to 3-fold across S-phase [48]. It is possible that nucleotide pools may begin to grow at an earlier point in the ring stage of the *Plasmodium* cell cycle, and it will be interesting to explore whether all the rounds of DNA replication occur at the same speed during schizogony, or whether later genome replications are completed more rapidly.

DNA damage responses, including DNA repair, cell cycle checkpoints, and cell cycle arrest and recovery phenomena can also be studied using BrdU labelling. This is particularly pertinent in light of recent reports that parasites with tolerance to the anti-malarial drug artemisinin rely on cell cycle arrest and ‘dormancy’ to survive [49]. As yet, the full molecular mechanism for this remains uncharacterized. Beyond responses to anti-malarial drugs, *Plasmodium* parasites may also have evolved replicative responses to many other stressors encountered in the human host. Dynamic host-parasite interactions remain understudied in human malaria parasites, yet they may have fast, flexible impacts upon virulence and disease pathogenesis. High parasitaemia, which is very likely to be partly determined by the rate of parasite growth and replication, is one of the best clinical predictors of severe malaria [50], so a better understanding of the factors determining parasite growth and replication could have real medical relevance.

As demonstrated here, the unusual toxicity of BrdU in TK-expressing *P. falciparum* parasites does not preclude its potential usefulness as an experimental tool, but does highlight another interesting way in which *Plasmodium* biology differs from that of model eukaryotes. It will be interesting to examine whether other *Plasmodium* species are more or less sensitive to BrdU when TK is expressed, and whether this correlates with any particular factor in their biology. For example, the A/T bias in genome composition of the mouse malaria species *P. berghei* is similar to that of *P. falciparum*, whereas that of the simian and human-zoonotic species *P. knowlesi* (which is also genetically tractable) is only ~62 %.

Overall, this innovation will open up many new avenues for studying the dynamics of DNA replication in the unusual cell cycle of *Plasmodium*. Since the biochemistry of all sequenced *Plasmodium* species appears to be similar, the potential is not limited to *P. falciparum*, but could be transferred to any *Plasmodium* model species amenable to transfection. This includes mouse malaria which can be studied inside the mosquito and mammalian hosts more easily than the human parasite *P. falciparum*. Since in vivo BrdU labelling in mice is already established, it would potentially be feasible to study all stages of the parasite’s lifecycle in these model systems. Finally, it will now be possible to explore the experimental potential of other thymidine analogs that will become viable tools in this new system, such as CldU, IdU and 5-ethynyl-2′-deoxyuridine (EdU). EdU is detected via click-chemistry rather than antibodies, opening up a different suite of new experiments in *Plasmodium* biology.

## Conclusion

The technique reported here will allow BrdU labelling of replicating DNA to be used in any *Plasmodium* parasite transfected with a TK gene. This opens up many new avenues for studying the dynamics of DNA replication in the unusual cell cycle of this parasite.

## Additional file

10.1186/s12936-015-1014-7 Controls demonstrate that BrdU sensitivity of TK-expressing parasites is not artefactual. A. qPCR data showing the average copy number of the TK gene in 3 different +TK parasite lines: +TK and +TK(B) shown in Fig. 3, and +TK(int) shown in Additional file 1C. Relative copy number is calculated relative to two single-copy housekeeping genes encoding seryl-tRNA synthetase and actin. Error bars show standard deviation of triplicate readings. B. MTS assay on the MCF-7 human breast cancer cell line, over a range of 1 mM to 3nM BrdU. Error bars show standard deviation of triplicate readings. C. MSF assay on the +TK(int) parasite line grown in the presence or absence of WR99210, over a range of 5 mM to 2.3 nM BrdU. Error bars show standard deviation of triplicate readings. IC50 values, calculated with GraphPad Prism software, show that there is no difference in the parasites’ sensitivity to BrdU in the presence or absence of WR99210. 95% confidence intervals are given in parentheses.

## Abbreviations

BrdU: bromodeoxyuridine
TK: thymidine kinase
CDKs: cyclin dependent kinases
ELISA: enzyme-linked immunosorbent assay
MSF assay: malaria SYBR Green I fluorescence assay
RCN: Relative Copy Number
MTS: 3-(4,5-dimethylthiazol-2-yl)-5-(3-carboxymethoxyphenyl)-2-(4-sulfophenyl)-2H-tetrazolium
DAPI: 4′,6-diamidino-2-phenylindole. h*DHFR*: human dihydrofolate reductase
IC50: 50 % growth-inhibitory concentration
*Pf*DHFR-TS: *P. falciparum* bifunctional dihydrofolate reductase-thymidylate synthase enzyme
EdU: 5-ethynyl-2′-deoxyuridine
h: hours
