# Supplementary figures and images for: Transfection with thymidine kinase permits bromodeoxyuridine labelling of DNA replication in the human malaria parasite Plasmodium falciparum
Source: Malar J. 2015 Dec 2;14:490. doi: 10.1186/s12936-015-1014-7 (PMC4668656; doi:10.1186/s12936-015-1014-7)

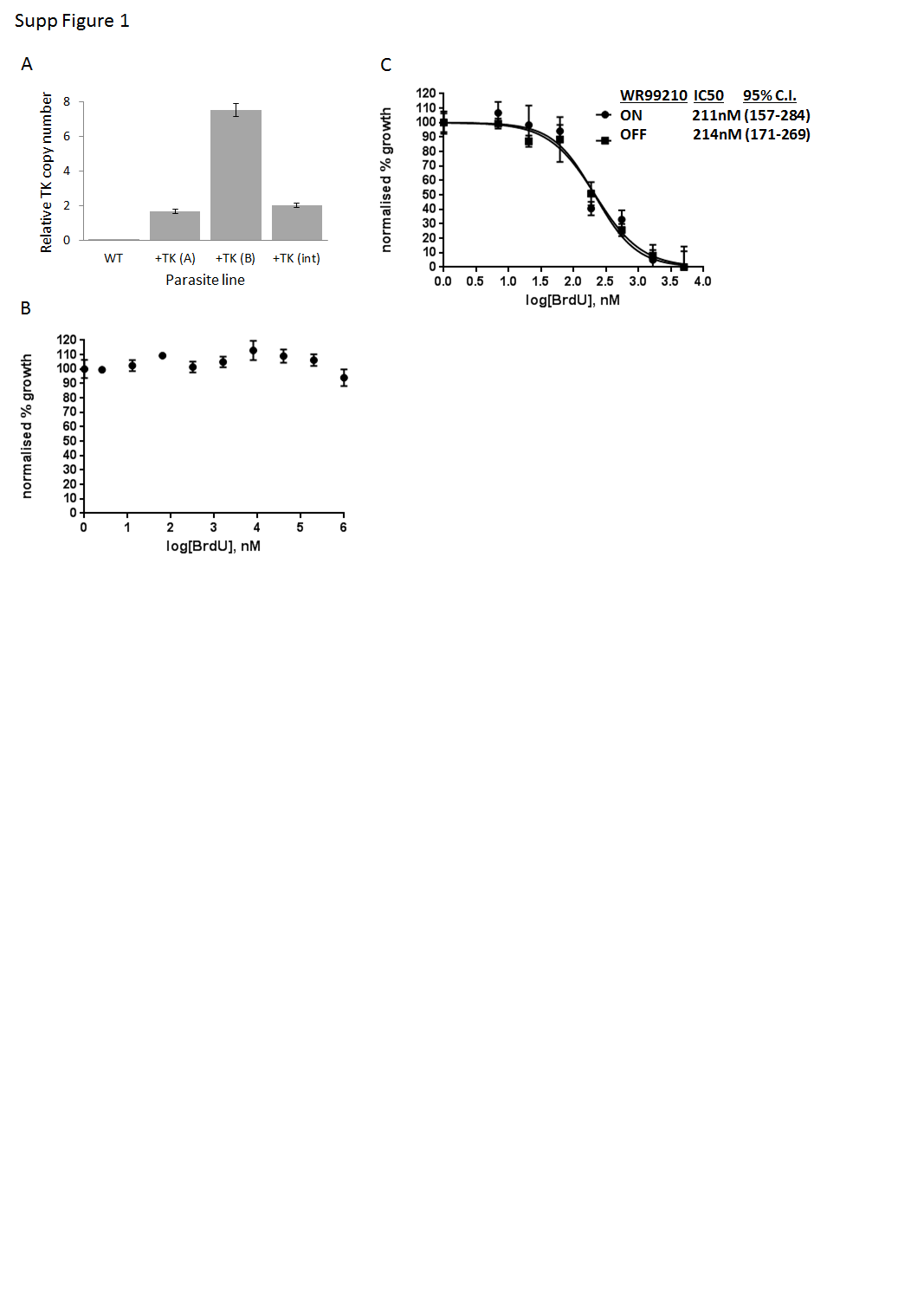

Supplement: Supplementary file 1 — 10.1186/s12936-015-1014-7 Controls demonstrate that BrdU sensitivity of TK-expressing parasites is not artefactual. A. qPCR data showing the average copy number of the TK gene in 3 different +TK parasite lines: +TK and +TK(B) shown in Fig. 3, and +TK(int) shown in Additional file 1C. Relative copy number is calculated relative to two single-copy housekeeping genes encoding seryl-tRNA synthetase and actin. Error bars show standard deviation of triplicate readings. B. MTS assay on the MCF-7 human breast cancer cell line, over a range of 1 mM to 3nM BrdU. Error bars show standard deviation of triplicate readings. C. MSF assay on the +TK(int) parasite line grown in the presence or absence of WR99210, over a range of 5 mM to 2.3 nM BrdU. Error bars show standard deviation of triplicate readings. IC50 values, calculated with GraphPad Prism software, show that there is no difference in the parasites’ sensitivity to BrdU in the presence or absence of WR99210. 95% confidence intervals are given in parentheses. [file 12936_2015_1014_MOESM1_ESM.png]
